# A Dissertation on the Effects of Malaria, and Other Malign Atmospherical Influences, in the Production of Febrile Diseases of Various Types and Degrees of Intensity; with Particular Reference to the Important and Specific Functions of the Ganglionic Nerves in the Phenomena of These Disorders: The Whole Being Intended to Illustrate Their Treatment on Physiological Principles

**Published:** 1832-12

**Authors:** Andrew Blake

**Affiliations:** Physician to the General Lunatic Asylum near Nottingham; late of his Majesty's 7th Regiment of Dragoon Guards, and author of "an Essay on Delirium Tremens," &c.


THE LONDON

### Medical and Physical Journal.

406, VOL. LXVIII.] DECEMBER 1832. [78, *New Series*.

"For many fortunate discoveries in medicine, and for the detection of numerous errors, the world is indebted to the rapid circulation of Monthly Journals; and there never existed any work to which the Faculty in Europe and America, were under deeper obligations than to the 'Medical and Physical Journal of London,' now forming a long but an invaluable series."—RUSH.

### ORIGINAL PAPERS.

### EFFECTS OF MALARIA, &c.

*A Dissertation on the Effects of Malaria, and other Malign Atmospherical Influences, in the Production of Febrile Diseases of various Types and Degrees of Intensity; with particular Reference to the important and specific Functions of the Ganglionic Nerves in the Phenomena of these Disorders: the whole being intended to illustrate their Treatment on Physiological Principles.* By ANDREW BLAKE, M.D., M.R.C.S., Physician to the General Lunatic Asylum near Nottingham; late of his Majesty's 7th Regiment of Dragoon Guards, and author of "an Essay on Delirium Tremens," &c.

WHEN the rudiments of a new being are projected in the formation of an ovum, they are endowed with the principle of animal life, although in a comparatively low degree of developement, in which condition they remain until the appropriate stimulus calls the vital spark into that activity which it afterwards displays, or until it be destroyed either by disease or by the death of the individual in whom the ovum originated.

This principle of life, although immaterial in its nature, is destined to sojourn in and endow its earthly tenement with vitality but for a given period; after which it is incapable of resisting the general law of nature, which ordains that all earthly things must, sooner or later, change their state of aggregation. We observe the inscrutable principle of life in a state of prodigious activity during the earlier part of our existence, displaying powers evinced by the rapid increase of the body, sufficient to induce us to suppose it as durable as the immaterial source from which it springs; but, as age advances, its energy and capability to resist ordinary chemical laws gradually diminish, and in a very few years cease altogether. While the vital principle maintains its governing power over matter, its particular attribute is that of being enabled to substitute the laws of what is termed animal or organic chemistry for those which are known to regulate the action of dead or inorganic substances one on another: this influence increases in the direct ratio of the general vigour of organic life, and vice versâ. It would appear that organic and inorganic chemistry are directly opposed to each other; the one tending to maintain the integrity of vital organization, while the other as constantly disposes to its destruction, and ultimately triumphs in the resolution of all bodies into their primitive elements.

From this view we perceive that the living being is assailed, from the first dawn of its formation, by the analyzing hand of that greatest of all chemists, Time, into whose unrelenting crucible all material bodies must at last enter, whether they possess organic or inorganic existence. The more perfect animals are, nevertheless, so constructed as to be enabled to set up actions capable of resisting this destructive agent for a certain length of time, longer or shorter according to the vigour of the individual. The human fabric appears, for this purpose, to be composed of two great systems, namely, the nervous and the vascular. The first comprises the brain and nerves, while the vascular system includes the heart and blood-vessels. These two great supporters of life depend reciprocally on each other, and are influenced by their respective conditions: thus, when they are both in a state of vigour and equilibrium as to energy, the highest health is the result. We are now supposing a case in which both the nervous and arterial systems are in the most perfect state of tonicity; in which state, unfortunately, the body seldom continues for any length of time: nature has been so indulgent, however, as to admit of our enjoying various grades of health, from that which has been just alluded to, and which is occasionally displayed by the vigorous European peasant, to that observed in the degenerate Creole of a tropical climate, or even in the Cretin himself, according to the circumstances under which the individual may have been placed. As long as the equilibrium of vigour is maintained, however delicate the being may appear, or however low the scale of health he may enjoy, he feels, with regard to his internal sensations, a degree of comfort, and is, to a certain extent, unmolested by acute disease; but so soon as any deviation takes place from this state of equilibrium between the two great systems, disease is the immediate consequence, and that generally in violence proportionate to the previous vigour of the habit.

The brain and nervous system are allowed by all physiologists to exert a most powerful influence over the rest of the body, conveying to every part of it that capability of action and sensibility which are necessary to its various functions; and in this respect their influence stands pre-eminent in the animal machine. They seem likewise to be the great medium through which we communicate with external objects; and, while they are themselves influenced in a particular manner by the state of all surrounding elements, they receive internal support from, and are acted on by, the contents of the vascular system, on the nature and condition of which their energy of action particularly depends. Thus we see the vascular system, on the one hand, depending on the state of the brain and nerves for sensation and capability of action; while, on the other, the nervous system owes its vigour not only to the state of external causes, such as the purity of the atmosphere, &c., but likewise to the healthy nature of the contents of the blood-vessels, or, in other words, to the wholesome condition of the blood; to the formation and perfection of which all the other organs of the body are subservient; some to its supply, as the organs of mastication, deglutition, digestion, and absorption; and others to its purification, by depriving it of principles, which, having served the principles of life, have become either noxious or useless: these are the lungs, the liver, the kidneys, the skin, and the various mucous surfaces, &c. When we recollect that the nervous system is influenced by all surrounding media, such as the atmosphere, &c., and that on its vigour depends the permanent capability of action in every other part of the animal machine, we cannot be surprised at the depressing effects which malaria exerts immediately on the brain and nervous system, and mediately on the vascular system and all its subsidiary organs. We are prepared to expect that all foreign matter capable of combination with the atmosphere, and of a nature hostile to the constitution, will prove a very fertile source of disease: of these there may be many kinds, some in the form of exhalations from the earth's surface, others in that of specific animal poisons. None, however, is more generally observed, or of more universal prevalence, than that which is known under the term malaria: of its specific nature in an uncombined state we know too little to be able to say more than that its production is connected with the decay and dissolution of vegeto-animal matter; and that, according to the different circumstances of temperature, and perhaps electric influence, under which the process takes place, its malign effects on the human fabric are more or less manifested by an immediate depression of the energy of the nervous power, and mediately by decreased action in the vascular system and all the secretory organs. This effect is so apparent and so well known to medical observers, that it needs no comment with regard to the fact itself; but in treating of the febrile diseases which are its offspring, their attention cannot be too closely directed to the important functions of that division of nerves denominated the central or ganglionic system; an admirable provision of nature destined to govern automatic life, and thus render it almost an independent power, possessing its own instinctive laws; though, if unassisted, it must ultimately either sink or become again subservient: it will be seen that, without this admirable arrangement, the whole fabric must have more frequently yielded to the overwhelming influence of those deleterious agencies which so often emanate from the surface of the earth, and which, when evolved in sufficient dose, prove deadly poisonous.

The ganglia would seem to be deposits of that species of instinctive nervous influence which is necessary, on any emergency, for the government and performance of the vital functions; they are not, like the brain and other parts of the nervous system, generally influenced by ordinary external lædentiæ, and are hence, owing to their independent nature, enabled to support life for a certain period, even under extensive injuries of the brain itself; during which time is allowed for its recovery from the effects of whatever may have assailed it, as apoplexy, wounds, &c.

The ganglia are to the nervous system what the "maintaining power," or "going fewsea," is to a watch; without it the works stop their movement while it is winding up. So it is with the animal system; a very slight injury of the brain would prove instantaneously fatal, by depriving it of nervous influence, were it not for the maintaining power of the ganglia. They are likewise endowed with a still more essential property, and one which manifests itself in all paroxysms of fever, that is, their capability of setting up (while the nervous power, properly so called, may be much depressed,) an increase of action in the vascular system, either locally or generally, according to their causes.

This increase in the action of the vascular system is instituted with a view to restore the loss of equilibrium between it and the nervous system, caused by the sudden depression of the latter, while the former remains comparatively unimpaired. George Hamilton Bell, in his "Treatise on Cholera Asphyxia," says, "the source of the sensorial and voluntary power is in the cerebrum and cerebellum; that of the respiratory power, in the upper portion of the medula spinalis; and that of the circulatory power in the ganglionic system of the great sympathetic."* On this principle we can explain the phenomena of almost all diseases of general increased action. Thus we see the healthy European soldier arrive in a state of vigour in a tropical climate, where malaria is always in existence to a greater or lesser extent; soon after which he may be attacked with violent symptoms of ardent fever, or what some authors have termed synocha. The intensity of these symptoms in this case is evidently owing to the vigorous efforts to restore the equilibrium which previously existed between the ganglionovascular and nervous systems in a sane and vigorous European.

The nervous power is generally diminished in an eminent degree by the influence of that most depressive agent, malaria; while the ganglionic system, or that portion of the nervous system which is not destined to communicate directly with external objects, but to carry on and regulate the instinctive or lower operations of animal life, is not equally effected by it. When, however, it happens that, in consequence of the operation of any morbific principle on the brain and nerves, the energy of the vascular system is so far impaired as to prove injurious, the ganglionic nerves are, by a law of their system, aroused to such an exercise of their specific functions as to display that independence of action with which it is so admirably endowed: in doing so, it induces all the symptoms of increased

* Vide Bell's Treatise on Cholera, p. 68.

arterial action which is so alarming and generally fatal, within the tropics, to those who are termed new comers: this effort, or febrile action, continues or remits, or is repeated in the form of paroxysms, according to the nature of the occurrence, either until death terminates the struggle, or until convalescence is induced; in which latter case we always find, at least for a given time, the scale of health of the individual much reduced from what it was previous to the invasion of the disease; for, although the great systems alluded to may have returned to a perfect state of equilibrium of vigour, yet neither of them can possess that strength or tone which they previously enjoyed. Should, however, this supposed vigorous subject arrive in the West Indies, for example, at a very healthy season of the year, when malaria is not evolved in sufficient quantity to induce that sudden depression of the nervous power which is necessary to alarm the ganglionic system, and lead it to excite fever, there will be, nevertheless, a sufficiency of it at all times in tropical climates to act in a slow and insidious manner on the frame universally, without, as it were, alarming the gangliono-arterial system; but, at the same time, not without depressing the general nervous energy so much as to influence, in common with all the functions, the process of sanguification, &c. While the nervous system, which is the prime mover of all living actions, is thus slowly and insidiously acted on by this destructive agent, and hence all the functions and secretions are affected and vitiated, the blood retains in a greater degree the principles which a more vigorous action would have eliminated from it; the lungs do not decarbonize it with their wonted energy, nor do the liver or kidneys perform their functions with their usual vigour: under such circumstances, the nature of the circulating mass become less stimulating to its vessels, and in this way their action and tone gradually and insensibly diminish, accommodating themselves, as it were, to the level of the already lowered nervous powers, and thus re-establishing the lost equilibrium between these two great systems; although, perhaps, as I have already stated, on a very reduced scale of vigour in comparison with that which the individual enjoyed on his first arrival within the sphere of malaria. This lowering of the scale of health, either through the more violent instrumentality of ardent fever, or the milder or almost unsuspected influence of diluted malaria, although destructive of physical force, is productive of certain advantages, by rendering individuals less obnoxious to the acute diseases of the climate; it is a tribute which all must pay who are doomed to reside in tropical latitudes, but by it they become what is termed seasoned or creolized, and may, for many years afterwards enjoy a comparative immunity from tropical disease, or, at most, be only susceptible of being affected with the mere chronic forms of it, such as dyspepsia, derangement of the liver, intermittent fever, &c.; while new comers, or those who have not passed through the necessary ordeal, may suffer from ardent fever of the most fatal description. It would appear that malaria exercises its debilitating influence, and acts on the nervous system in the direct ratio of the state of vigour enjoyed at the moment of its application: hence, medical men will be struck by the apparent anomaly of a healthy European soldier being a prey to the result of a debilitating cause, such as that just alluded to, while the more feeble and debilitated old resident, or creole, will escape its deleterious ravages; but experience teaches us that such is the fact, however contradictory it may appear. The dose of malaria which will act on the unassimilated constitution having little or no effect on the seasoned resident, the gangliono-vascular systems of the former being in that state of vigour which disposes them to violent reaction on the slightest depression of the nervous system; while, in the seasoned residents, all the powers have been already so reduced as to render them less susceptible of the influence of miasmata; diarrhœa, dysentery, cholera, ague, or disease of the liver, being the result in them, according to the existing predisposition.

We have thus shewn the general law of predisposition, resulting from the different circumstances in which the subject is placed; but of the peculiar and very occult conditions prevailing in the habits of individuals, and conveying a greater susceptibility to one kind of malign influence than another, we must acknowledge our ignorance, as well as of many other inscrutable circumstances connected with life itself.

Although malaria is not cognizable to our senses, we have sufficient evidence of its effect in the production of disease, even under ordinary circumstances, and we are not without examples of its effects being so overwhelming to the entire system as to be productive of almost immediate extinction of the vital powers; particularly if the subject be not snatched from his threatened fate by the operation of diffusible stimuli of the most powerful nature, aided, perhaps, by the cautious abstraction of blood, to relieve that state of congestion which arises from extreme debility. In these cases the destructive agent must be so intense as to annihilate, as it were, at the same moment, the vigour of the nervous and ganglionic systems, and thus deprive nature of the power of making an effort to relieve itself. These effects resemble asphyxia induced by the inspiration of carbonic acid gas: this deleterious gas, however, acts more directly, in the first instance, on the blood, by not affording the lungs an opportunity of eliminating carbon from it; in consequence of which, the vital fluids become incapable of supporting animal life, and death, without the least reaction, is the result.

The constitution which has survived the first attack of fever, or has become creolized by the gradual influence of malaria on the nervous system, and, through it, on the vascular contents, will only be subject to fever of a remittent or intermittent form; while the vigorous "Johnny Raw" will oftentimes be carried off in a few hours or days by ardent fever. In the former case, the reduced system is incapable of making continued efforts of a sthenic nature, and is therefore compelled to be restricted to those of a remittent or intermittent type, which may be protracted to an indefinite extent. Fevers, and all paroxysmal diseases, in our own country, are induced by the same influence as those which occur within the tropics; but they are rarely so obstinate or fatal, owing to the modification of the causes, as well as the difference of constitution which our climate induces: but when what is termed typhus fever rages, although, for obvious reasons, it generally prevails amongst the poorer classes, we find that, when it succeeds in obtaining admission into the mansions of the rich, it is comparatively more fatal, considering all the circumstances with regard to the condition, and consequent means of resisting disease, which the rich possess, compared with those which the lower orders can command. This comparatively greater mortality must arise, in the first instance, from the more violent reaction which the vascular system is capable of making, at least for a short time, and subsequently to the depression of the vital powers which succeeds to the sudden privation of high living. We find, then, that malaria acts as a direct sedative on the brain and nervous system generally, and would, if not prevented by the wise provision of nature, which erects the central or ganglionic system into a sort of independent or preservative power, be destructive of the vital energies, and afford daily examples of death following exposure to such debilitating causes: this preservative power is manifested by efforts made in the form of paroxysms, occurring once, or oftener, according to circumstances, until the equilibrium already alluded to be restored. During these paroxysms, local determination may be induced, either in the brain; according to Clutterbuck's theory of fever; in the lining membranes of the digestive organs, inducing what is called Gastro-enteritis, according to the opinions of Broussais; or in the venous system, causing the congestive stage of Armstrong. Celsus says, "we have each of us our weak point;" to which the peculiar action or determination may be directed, inducing inflammation, cholera, dysentery, affections of the brain or chest, &c., according to individual predisposition and the intensity of the causes.

When the equilibrium between the nervous and vascular systems is once destroyed by the sudden depression of the nervous power, the first result is general debility and coldness of the surface. The natural consequence of this state is accumulation of blood in the interior, arising from the want of energy in the system to project it to the surface: thus the ganglionic or automatic system becomes stimulated by the distention of the larger vessels, over which it has a direct influence; and in its efforts to rid them of the unusual *gêne* under which they labour, the symptoms of fever are developed, assuming either the intermittent, the remittent, or the continued form, according to the intensity of the causes and the previous grade of health of the individual affected. Should the individual attacked enjoy a high degree of vigour at the moment of his exposure to morbific causes, the result will be proportionably violent or dynamic. The nervous debility, or what is termed the cold stage, will be longer or shorter according to the dose or intensity of the malaria; and the reaction, or hot stage, will likewise be influenced by the same causes, as well as by the vigour of the system. Should, however, the scale of health have been previously reduced by long and gradual exposure to miasmal influence, the vascular and nervous systems will have kept pace in their loss of vigour; owing to which circumstances, the equilibrium between these great systems will not have been so much interrupted. During such a change, however, the lungs, liver, and kidneys participate in the general diminution of energy, and cease to secrete from the blood with their wonted vigour those principles which it was their office to deprive it of: hence this vital fluid becomes unfit to stimulate its vessels in a healthy degree, so that every part of the system sinks gradually, but "*pari passu*," without exciting any immediate effects beyond those attendant on a general decrease of energy; though, after a short time, the liver and digestive organs may become particularly affected, and their derangement may be only the precursor of a train of diseases depending on dyspepsia, which are well known to the medical world generally, but more particularly to those whose lot it has been to reside within the tropics. While the scale of health is thus reduced generally by the slow action of morbific agents, although the individual exposed to them may appear to enjoy an immunity from ardent fever, yet any great increase of their intensity will, by acting suddenly on the nervous system, destroy even this lowered equilibrium, and induce a series of symptoms proportionate to the relation, to the intensity of the cause, and vigour of the system. Thus, should the lungs have had their vital powers diminished, and consequently their decarbonizing influence on the blood lessened, while the liver eliminates bilious principles from it in a smaller quantity than usual, and the kidneys carry off less of the superabundant azot, we must expect to find that the blood will have become surcharged with noxious principles, and cease to afford the necessary stimulus to the system generally; and, consequently, it cannot have the effect of exciting those continued efforts which are characteristic of inflammatory fever; while the tendency to putrescency which is manifested by the nature of the fluids generally, owing to the retention of the noxious principles alluded to, must fit them for that typhoid type which distinguishes all constitutional efforts under such circumstances. When the system is exposed to morbific causes of a highly concentrated nature, as is the case in the production of cholera, asphyxia, or malignant tropical fever, the general nervous depression is so great as to render it incapable of efficient reaction, and death, in many cases, is speedily the consequence, unless it be averted by the immediate and appropriate application of remedies to relieve congestion, and stimulate the system generally to re-establish the equilibrium of the circulation.

*Highly concentrated causes* will, of course, act on all living beings, but they will do so with more violence on the weak and broken-down constitutions of the poor than on persons within whose reach are the common comforts of life, and who, generally speaking, do not live so much within the sphere of the morbific causes. We know, from experience, that those who pass their days and nights in low and filthy situations, and whose constitutions are predisposed to disease by poverty and its consequences, must be the first victims to those emanations which some unknown influence, perhaps electrical, causes to emanate from such situations: causes of this nature, which are sufficiently intense, may induce death within a short period in constitutions so prepared; while, in a previously healthy subject, they might only be productive of simple cholera, dysentery, or fever.

A retrospect of what has been said will explain how malaria, when applied to the newly-arrived European in a tropical climate, may so suddenly depress the nervous energy, and thus destroy the equilibrium of the system, as to call forth the preservative efforts of the ganglia, and cause them to react and induce fever; while the same causes would scarcely have had any effect on a system already lowered by gradual exposure: hence the comparative immunity of Creoles and old residents from febrile affections, except when exposed to very concentrated causes; and even then they are rarely assailed by ardent fever, while they are victims to bilious remittent fever, typhus icterodes, cholera, or ague. We observe that the brain cannot perpetually secrete sensorial power: hence sleep is ordained, to enable it to recruit, during the night, that energy which it expended by day. We see the ganglionic system subservient to the same laws, being in itself incapable of continued action: it is, therefore, obliged to have recourse to a similar mode of alternate action followed by comparative repose, as is exemplified in fevers by remissions or intermissions. It may be asked, how does the ganglionic system continue its action without apparent cessation, as is manifest by the continued action of the lungs, heart, arteries, and digestive organs, during sleep, or (what is more extraordinary) under the influence of apoplectic or other pressure on the brain? The reason is this: although the ganglionic system is so far independent of the general nervous system as to be capable of supporting vital action for a given time, under the circumstances alluded to, and of even taking on increased action in cases of fever, it is incapable of doing so long; and, were it not for a beautiful arrangement of nature, it too must have slept, during which the vital functions would have been arrested; but the ganglia appear to be depôts or reservoirs of nervous influence, which is perpetually supplied to them while the cerebrum is not under the influence of sleep, and is in a healthy state; so that the store of it collected in these nervous bodies becomes so great as to enable the vital functions to be carried on with the necessary vigour during the interval of cerebral repose; or they may also, for what we know on the subject, have the power of secreting to a certain extent the influence necessary to govern instinctive action, and thus, by furnishing in part this essential influence, be enabled to maintain vital action, under all the circumstances already detailed. During sleep, however, all the instinctive functions are carried on more slowly than when awake: even during the first few moments after the cessation of sleep, we are conscious of a dulness and want of vigour in our system, exemplified by yawning and stretching, which continue until the sensorium has once more replenished the ganglia, and restored them to vigorous action. While asleep, the pulse becomes slow, and febrile action diminishes, the vascular system during that state being only influenced by the ganglia; while the pulse becomes quicker towards evening, or when it has been for a certain period governed by the united influence of the brain and ganglionic system: hence, febrile exacerbations occur towards the close of the day. By this wonderful arrangement, we observe the vital functions continued even when the brain is suffering from compression; but they cannot go on long under such circumstances. The brain being deprived of the power of contributing to supply the ganglia, if the pressure be not removed, their resources become exhausted, and death speedily ensues. For the same reason, we find pure continued fever is but of short duration; and, should health not succeed to it, the ganglia become unable to support long-continued action, and a change is effected in the form of the fever, from continued to that of remittent or intermittent. Mr. M'Dermott says, "The ganglia of the sympathetic, small, nervous, or cerebral masses, devoid of perception and intellectual faculties, have their own organic instinct or functions, whereby certain vital qualities are developed in them, imparting the necessary sensibility and involuntary action to the vital viscera; whilst they unite the latter in sympathy with the cerebro-spinal system."*

By this explanation we are taught that the ganglia are capable of exercising their peculiar functions, independent of volition; while it admits of their being joined in sympathy with the brain and nerves of voluntary motion. This sympathy is clearly evinced by the increase of action in the heart, arteries, and respiratory organs, which follows passions of the mind, &c. We can also explain how the ganglia are capable of supporting continued action in the respiratory, vascular, and digestive systems, by taking into consideration the circumstances of their being united "in sympathy with the cerebro-spinal system." The ganglia are not less subject to the laws which govern all nature than the other parts: we observe the brain and nervous system obliged to repose from their labours, in order to receive or secrete a fresh supply of the elements of what is termed nervous influence: hence all animal and vegetable life sleeps, for a longer or shorter period, as their peculiar structure

* Vide Lancet, No. 420, vol. ii., 1830-31, p. 776.

may require to recover from the exhaustion which the labours of life induce. GREGORY says,* "Homo non semper valit ad sensum motumque voluntarium exercendum: utramque functionem oportet per intervalla feriari, ut vires musculorum nervorumque, exercitatione exhauste, quiete et olio reficiantur." With a view, then, to enable certain indispensable functions to preserve their state of activity during sleep, as well as while awake, the supreme Architect, in his wisdom, devised the ganglionic system; each ganglion being a reservoir or depôt of nervous influences, is supplied with it from the cerebro-spinal system in sufficient quantity to provide, not only for the current exigencies of the body while awake, but likewise to furnish a store to enable the sympatheticus maximus to govern the vital functions for a given period during sleep, or even under injuries of the brain and spinal marrow themselves. We have here an instance of that exquisite adaptation of the means to the end, so beautifully exemplified in all the works of nature, by which the purpose is fully accomplished, without any waste of her resources; for we find that this store of nervous influence is only subservient to instinctive or involuntary functions, as these are all that require to be kept in action under the circumstances alluded to. Thus we see that, when the brain is compressed from apoplexy or other causes, its functions are suppressed as long as that state continues; during which suppression the ganglionic system is capable, from its independent endowments, of continuing to govern the vital functions for a given time, either until the pressure be diminished or removed from the brain, or until the ganglionic state of nervous energy be exhausted; in which latter case, death is the inevitable result. When the brain itself is engaged in making what may be termed cerebro-paroxysmal efforts, as in delirium tremens.† Sleep does not take place during their continuance, which is oftentimes for several days; and when that much-desired event does occur, it is the signal of convalescence. Now, as we see the use of the ganglia, and their intimate connexion with the nervous system generally, we must perceive the great sympathy which exists between them, and hence be enabled to explain how depressing causes, being applied to the system in a gradual manner, will weaken insensibly the nervous and ganglionic powers at the same time, so as to lower the scale of health generally, without materially deranging the necessary equilibrium between them; while we can also perceive how the sudden depression of the nervous power will have a contrary effect, the ganglionic system, being less obnoxious to the direct influence of external agencies, will comparatively retain its vigour, and be capable of making instinctive efforts, in the form of increased action, to raise the vigour of the brain, with a view to the re-establishment of the lost equilibrium between these two essential branches of the animal economy.

* Conspectus Medicinæ Theoreticæ.

† Vide Essay on Delirium Tremens; 1830.

When the general scale of health has been previously so lowered as to render the system incapable of making sthenic efforts, the sudden application of depressing agents of a more concentrated nature than those to which the body has been hitherto accustomed, may be productive of intermittent fever; one or more paroxysms of which may be the consequence. At the termination of each, apyrexia succeeds, and continues for a longer or shorter period, according to the degree of vigour which may have existed at the moment of the attack; and this vigour will always be found to depend on the healthy condition of the blood. During the existence of a paroxysm, we observe a regular succession of symptoms, all wisely ordained for the restoration of health, coldness, and debility: the natural effects of sedative applications are attended with nausea and vomiting, which efforts we find tend, in an eminent degree, to re-establish the circulation, and at the same time to emulge the hepatic vessels, and thus rid the blood of superabundant bilious principles. During the hot stage, we observe the blood propelled to all the secretory organs, with a view to the restoration of vigour and the reparation of the injury offered to the system; and, lastly, we see the febrile heat and excitement gradually and safely diminished by profuse perspiration, which not only prevents topical determination, and carries off excess of caloric, but furnishes extensive means of evacuating impurities from the blood, and at the same time of lowering the vigour of the gangliono-arterial system towards the level of the depressed nervous power: in a word, we recognize the hand of the Allwise by a wonderful combination of efforts employed, almost at the same moment, in raising the depressed energy of the nervous system, in lowering the powers of the vascular system, and in improving the quality of the vital stream, so as to reestablish equilibrium between these essential systems, at the least possible risk to the integrity and well-being of the human fabric.

We have observed that ague will not attack a healthy subject immediately on being exposed to miasmal influence: exposure in such a case might be followed by inflammatory fever; but, if the same subject should have been exposed to the gradual influence of miasmal exhalations, the entire fabric would have become insensibly affected, the cerebro-spinal system slowly lowered, and with it the vigour and condition of all the functions of the body. The respiratory organs, as I before stated, decarbonize the blood with less energy; the liver secretes or eliminates a lesser quantity of bilious principles; the action of the kidneys, with regard to the separation of urea, is also diminished; and the skin and digestive organs are proportionately affected in their particular functions: hence, the quality of the blood becomes essentially deteriorated, and unfit to support what may be termed the sthenic actions of life; the system, in consequence, is obliged, when assailed under such circumstances, to content itself with making preservative efforts of the asthenic kind, in the form of remittent or intermittent fever, according to the intensity of the causes and the health and vigour of the individual.

Should the causes be slight, ague may be the result; but in the event of their being concentrated, the constitution, however reduced in energy, will in general make a proportionate effort, and induce remittent or typhoid fever.

When these efforts of nature assume an intermittent form, they will manifest themselves in the form of quotidian, tertian, or quartan, &c., according to the vigour of the system and intensity of the causes; the quotidian requiring the greatest proportion of vigour in the system, the tertian next, and, lastly, the quartan. From what has been stated, it will appear evident that the vigour of the system depends on its original conformation, on the nature of its nutrition and habits, and, though last not least, on the influence of external agents, such as those applied to it through the medium of the atmosphere, and which act directly on the nerves, and, through them, on the secretory organs, and finally on the quality of the blood itself. If the conformation be vigorous, the food wholesome and in due quantity, and the circumambient atmosphere pure, moderate exercise will maintain the proportionate secretion from the various organs, and perfect health will continue as long as the divine hand of Providence has ordained we shall resist the destructive effects of time. "Homo enim quamvis sanus et robustus et vegetus, labentibus annis et ipse delabitur, senescit, moritur in pristina elementa solvitur, aliis animantibus pabulum futurus."*

Should any of these necessary conditions be wanting, the health must suffer in a proportionate degree: thus, should the sustenance be of an improper kind, the chyle will necessarily be unwholesome, and the quality of the blood altered; consequently, the solids cannot be duly supported, and a disease of a cachetic tendency will be the result. What is termed high living, on the contrary, will induce an opposite state both as to quality and quantity of the blood, producing what has been called Hyperæmia, with a disposition to inflammatory action; again, should the nervous energy be depressed by exposure to malaria, the whole system immediately participates in the effect; and, however appropriate the nourishment may have been, the processes subservient to the formation of healthy blood cannot be sustained, as the action of all the secretory organs becomes impaired; the lungs, the liver, the kidneys, &c. become less active in their respective operations, and hence cease to eliminate with their wonted energy those principles which they are destined to separate from the blood, the presence of which deprive it of those stimulating properties which are necessary to excite the solids and support the healthy tone of the body. Thus, we see how the healthy state of the solids and fluids reciprocally depend on and influence the respective condition of each other, and, hence we shall be enabled to settle the long agitated theories of humoralism and solidism, by adjudging to each its due meed of influence.

* Conspectus Medicinæ Theoreticæ, p. 3.

We have daily examples of scurvy and other asthenic diseases being the result of improper diet, which acts directly on the fluids, and indirectly on the solids; while malaria acts directly on the solids, the nerves for example, and indirectly on the blood, in the manner already described. This is all that appears necessary to be known on this subject, at least as far as practice is concerned.

I would not go so far as the learned Andral has done in his Pathological Anatomy: in speaking of an individual who breathed an atmosphere charged with miasmata, or fed on unwholesome food, and who became sick in consequence, this enlightened author says, physiology would lead us to infer that the blood, in such a case, "has been at least the vehicle of the morbific matter residing in the air or in the blood."* This is reviving, in toto, the doctrine of the humoral pathology, and is calculated to lead us to limit our practice to endeavour to evacuate the "morbific matter," which by the way is not appreciable in the blood, to the neglect of the more essential means, namely, those which tend to support the system in its efforts to re-establish the necessary equilibrium already alluded to.

The celebrated Cullen, in his Practice of Physic, says,† "While we thus reason against the notion of fevers being an effort of nature for concocting and expelling a morbific matter, we by no means intend to refuse that the cause of fever frequently operates upon fluids, and particularly produces a putrescent state of them. We acknowledge that this is frequently the case; but, at the same time, we maintain that such a change of the fluids is not commonly the cause of fever; that very often it is an effect only; and that there is no reason to believe the termination of the fever to depend upon the expulsion of the putrid matter."

The sentiments here expressed appear to be strictly correct: we know that an individual may be attacked with fever whose blood is in the greatest state of purity, as when synocha is induced; and that during the continuance of the disease, the governing powers of the solids being diminished, the fluids generally may degenerate in quality, and approach a state of putrescency which fits the system for asthenic efforts only. If, as I already stated, a man enjoying the highest state of health be exposed to miasmal influence, the great cerebro-spinal system will be particularly depressed in vigour; and through it all the functions of the body. The blood-vessels are particularly under the control of the ganglionic system, which is known to be gifted with powers, in a great degree independent of the brain, and to be capable, as the conservative power of the fabric, to cause the heart and arteries to display increased action in the form of fever, with a view to the re-establishment of the lost equilibrium between them and the cerebral system: during and previous to this state of disturbance all the functions are impaired, and hence the gradual change in the condition of the fluids, as al-

* Vide the analysis of M. Andral's work in the Medico-Chirurgical Review, No. 30, for October 1831, p. 343.

† Vide Cullen's Practice of Physic, vol. i. p. 43.

luded to by Cullen. In fact, this great author's reasoning on the nature of fever displays his wonderful observation, although it resembles certain hallucinations during which an individual argues soundly on false data. He sums up his opinion on the subject in the following words:* "Upon the whole, our doctrine of fever is explicitly this: the remote causes are certain sedative powers applied to the nervous system, which, diminishing the energy of the brain, thereby produce a debility in the whole of the functions, and particularly in the action of the extreme vessels: such, however, is, at the same time, the nature of the animal economy, that this debility proves an indirect stimulus to the sanguiferous system; whence, by the intervention of the cold stage, and spasm connected with it, the action of the heart and larger arteries is increased, and continues so till it has had the effect of restoring the energy of the brain, of extending this energy to the extreme vessels, of restoring, therefore, their action, and thereby especially overcoming the spasm affecting them; upon the removing of which the excretion of sweat, and other marks of relaxation of the excretories, takes place."†

This is an accurate description of a paroxysm of ague, so far as regards its cold, its hot, and its sweating stages; and it is correct with regard to what its author states concerning the remote causes of fever being of a debilitating nature; but it would be unnecessary for me, in these enlightened days, to attempt to combat the exploded doctrine of spasm in the extreme vessels, which this learned physician was obliged to create for the purpose of explaining the cause of reaction. It is much more rational and accordant with the laws of physiology to refer the reaction to the efforts made by a conservative power, which nature has destined to govern the action of the vital functions while the cerebro-spinal system may be either in a state of repose during sleep, or in a state of depression from exposure to miasmal influence, &c. This power we find to reside in the ganglionic system; and its efforts to re-establish the loss of equilibrium between the brain and vascular system, or what is termed fever, will, as already stated, be either continued, remittent, or intermittent, in proportion as the condition of the circulating fluids approaches or departs from the healthy standard.‡ Should it have been healthy when the equilibrium was interrupted, the fever will be sthenic or inflammatory; but should the subject have been previously exposed to the influence of malaria, or should the great secretory organs, such as the liver or kidneys, be func-

* Cullen's Practice of Physic, vol. i. p. 58. † Ibid.

‡ Professor Delpech, in his post-mortem examination of patients who died of cholera, thought he detected organic lesion in the nerves of the ganglionic system, and hence attributed the disease to the effects of this morbific condition of the ganglia. We are, however, aware how much functional disease may exist without any appreciable marks in nervous matter; a circumstance which would lead me to imagine that, whenever such phenomena were detected after this disease, they arose from contiguous sympathy, being situated immediately in the vicinity of the parts which suffered most from congestion.

tually or organically deranged from other causes, such as local disease or *improper food, &c.*, the blood will, under such circumstances, abound with those principles which, in a state of health, would have been eliminated from it; and it will, in consequence, cease to give that support and stimulus to the vessels which it was wont to afford them: hence the system becomes unable to make continued and sthenic efforts to relieve itself, and is obliged to be contented with those of an asthenic nature, in the form of remittent or intermittent fever.

It was observed that many of our soldiers who served in the campaign of Flushing in 1809, enjoyed tolerable health while on that duty, but were attacked with ague several months after their return home.* This curious fact is in accordance with the theory here propounded: the constitutions of such men at the time of their sejour in Walcheren, where they were exposed to malarial influence, were capable of resisting such influence so far as to maintain the necessary equilibrium of the systems, although the nervous power and the system generally may have been so far lowered as to influence the biliary secretions, &c., owing to which the blood became more or less charged with impure principles, and hence unfit to support sthenic action; thus the predisposition to ague was established, and the individuals alluded to, when in this country many months subsequent to their return, on slight exposure to febrile causes, suffered from intermittent fever; while other soldiers, who had not been thus prepared, escaped altogether; or were, perhaps, attacked with inflammatory fever. This predisposition to ague, and to all the various diseases which are the consequence of a residence in countries where malaria prevails, such as disorder of the digestive organs in all its protean forms, tic douloureux, &c. may remain, as it were, in a latent state for a long period, which is easily explicable on the above principles.†

We have, I hope, made it appear that the type of fever depends altogether on the condition of the circulating fluids in combination with the degree of intensity of the malarial influence at the moment of the rupture of the equilibrium of the functions, and that it will be sthenic or asthenic in its nature, according to these circumstances; continued fever being indicative of the greatest state of purity in the circulating mass, next remittent, and lastly ague or intermittent fever; and this last will be either quotidian, tertian, or quartan, as the causes may be intense and the fluids vitiated: should the causes be slight and the blood tolerably healthy, quotidian efforts would follow; but were they intense, and the blood impure, remittent fever of the asthenic kind would arise; while slighter

* We can confirm this fact from our own observation, as we had charge of several of the troops after their return from Flushing. Many of the men, who had previously enjoyed good health, were attacked with ague some months after their arrival in this country.—Editors.

† Vide Dr. James Johnson's excellent treatise on Change of Air; articles, Cretinism, Pellagra, and the effects of Italian Malaria.

causes with a similar state of the blood would, perhaps, be followed by quartan ague. As a general law, the violence of febrile efforts, whether sthenic or asthenic, will be in the direct ratio of the purity of the blood, influenced by the intensity of what are termed the febrile causes applied to the constitution, as well as by the length of time of their previous application. When speaking of the causes of fever we have, hitherto, referred them to malaria; whether it proceed from the decomposition of vegeto-animal matter in the form of swamp or filth, or from the more confined situation of a ship's hold;* but I do not thereby wish to exclude from our consideration contagion, which would appear to arise from the body during malignant febrile affections, such as typhus, the exanthemeta, &c., and has the power of propagating such diseases under particular circumstances from person to person. Atmospherical causes, from whatever source they may arise are, however, the primary agents in these cases, and contagion may then appear to become the immediate cause of their propagation. I must, at the same time, declare that, during a residence of six years in the West Indies, where it may be supposed I had ample opportunities of witnessing dysentery and yellow fever, with occasional cases of cholera,† amongst the troops and natives, yet I never could trace the origin of a single case to the effects of pure contagion; while, on the other hand, I had the most conclusive evidence of their endemial origin, as is detailed in my reports to Sir James M'Grigor, director general of the army medical department. It would be irrelevant to our present subject to enter further into this intricate and long-disputed question, and will suffice to state that all febrile causes, whether they arise from the surface of the earth or of the human body, are of a highly debilitating nature, and act by depressing the nervous energy in the first instance; which depression gradually extends to every organ of the animal machine, and, should fever not be the immediate consequence, deteriorates all the secretions; owing to which, the fluids become impure, and incapable of imparting healthy stimulation to the vessels of the system. We have a familiar example of this state in jaundice: in this affection the liver ceases to secrete bile in due quantity from the blood, which, in consequence, becomes impure, and incapable of acting as a healthy stimulus to the heart and arteries: hence the remarkable slowness of the pulse in ordinary cases of hepatic obstruction.

Having endeavoured to explain the phenomena of fever on simple anatomical as well as physiological principles, it will not, I

* Vide an interesting account of the cause of fever on board the Pyramus frigate, by Dr. Musgrave, of Antigua, in the Medico-Chirurgical Review for March 1824, p. 995.

† As cholera prevailed in this country during the greater part of the time devoted to the manuscript of this dissertation, I have ventured to give my opinions concerning its nature and origin in the form of a brief Appendix, with a view to the explanation of its phenomena as a febrile disease. See the end.

trust, be necessary for me to combat the great variety of ingenious, complicated, and in some instances visionary theories, which have been broached by physicians with regard to this important subject, from the time of Hippocrates down to the present day: such a proceeding would only take up time to no purpose. The learned Dr. Good, in his System of Medicine, when speaking of fever, says, "No complaint is so common as fever; none in which mankind, whether professional or laical, are so little likely to be mistaken, and yet none so difficult to be defined." While the celebrated Fordyce, in terms not less vague than general, says, "It is a disease of the whole system." After such authorities, it is with the greatest diffidence that I attempt to solve a difficulty which has evidently been a stumbling block to physicians of the first celebrity in all ages. In accordance with the principles laid down in this essay, I am, however, induced to define the disorder of fever to consist in a depression of the nervous energy, followed by increased functional efforts in the ganglionic and vascular systems, which assume an ephemeral, continued, remittent, or intermittent form, and may be accompanied or followed by perspiration, hemorrhage, eruptions, fluxes, local congestion or inflammation, and terminate either in convalescence, on the establishment of equilibrium of action, in some new form of disease, or in death.

We have here endeavoured to frame a definition which embraces all species of fever, although the object in writing the present paper is only to draw the attention of the profession to the peculiar part which the vascular system, under the influence of the ganglia, plays in their production; and, at the same time, to explain the deterioration which takes place in the nature and condition of the fluids of the body, from long exposure to febrific causes; such as malaria, even in a diluted form, or from the effects of an unwholesome nutriment taken for any length of time.

A due consideration of the theory expounded in this essay will, I trust, simplify the principle on which the *methodus medendi* is to be directed and modified in cases of fever. I also hope that it may tend to dispel the degree of uncertainty which hitherto embarrassed the practitioner, and is inseparable from the doctrines which have been promulgated on this subject. However successful a practice may be, it cannot be satisfactory unless its mode of action be understood, and capable of explanation on physiological principles: hence, although many theories and methods of cure have been proposed for these affections during the last two centuries, the empirical practice of the immortal Sydenham, which was that of "moderating action when excessive, and propping the vital powers when evidently sinking," has been found, by experience, to be that which is the most successful; and it is, perhaps, all that can be done even at the present day: such a practice will, however, be more successfully directed by the practitioner who can explain its rationale, as, by such a knowledge, he will be enabled to modify treatment with more certainty, and in many instances to anticipate nature in her operations, and thus assist her materially in her curative efforts: and this assistance is best afforded by carefully observing her course, and by endeavouring to imitate her, and, by so doing, aid her in her efforts to relieve the system when labouring under fever of either the sthenic or asthenic type.

Thus we observe that when any sedative, such as malaria or cold, &c., which acts by depressing the vital energy generally, be applied to the system of a previously healthy subject, the preservative efforts of the ganglionic system, should they not be rendered incapable of action by the intensity of the sedative, in which case death is the result, are in due time roused into activity in the form of increased vascular action of the sthenic order: the object of this will be to endeavour to raise the depressed powers of the nervous system, and at the same time, by the evacuations which are attendant on increased action, to lower the vigour of the vascular system, so as thus, by raising one power and lowering the other, to tend towards the restoration of equilibrium between both in the most direct way, and with the least possible injury to the constitution. Should these efforts succeed in re-establishing equilibrium, although both the nervous and vascular systems shall have suffered in the struggle, and both be lowered in the scale of their vigour from that which they previously enjoyed, yet the fever will probably have been only ephemeral, and convalescence will be the result: in such a case, venesection to an appropriate extent, in order to guard against local determination and structural disease, aided by the employment of means which increase excretion from the skin, the mucous linings of the stomach and intestines, and which at the same time emulge the biliary and urinary organs, are those which are indicated, and will, in the greater number of cases, prove successful. The evacuating plan of treatment is found to be most successful in cases of inflammatory fever; as, in the high state of vigour necessary to its developement, the system will bear evacuation with comparative impunity; while any attempt to re-establish the lost equilibrium by invigorating or raising the depressed nervous energy would, in all probability, increase the fever, and protract the disease. Should determination to the head, the lungs, or the mucous linings take place, a repetition of the depletory measures, both local and general, may become necessary; in addition to which, counter-stimulants and deobstruents, such as blisters and mercury, will be found efficacious. During the course of this disorder, it is likewise highly important that particular attention should be paid to prevent the accumulation of superabundant caloric; this is best effected by sponging the body. Should these means not succeed in arresting the progress of the disease, nature will proceed to make fresh instinctive efforts, which, although instituted with the best intentions, may prove highly destructive: "la puissance vitale conservatrice abandonnée à elle-même, qui, reposant uniquement sur les lois organiques du corps, n'agit non plus qu'en vertu de ces lois, sans raisonner et reflechir ses actes."*

The system appears to be governed, in her salutary efforts, by a series of general laws, the whole or some of which are called into action in every species of inflammation, whether local or general; and besides the influence they exert in the increase or decrease of the various secretions, with a view to re-establish equilibrium between the systems, they call to their assistance other active processes, some of which act diametrically opposite to each other in their immediate effects. In cases of local inflammation, the absorbents are called upon to act with a view to the removal of the offending cause, or at least to insulate it, so as to prevent its admission into the system; while in general inflammation, such as fever, the coats of the blood-vessels are oftentimes partially removed, so as to admit of hemorrhage, and thus relieve the existing plethora: at the same time we may observe all-provident Nature guarding against the injury which such a destruction of parts might cause, by the developement of the buffy coat in the blood, to enable her to deposit coagulable lymph wherever a solution of continuity may require its application. Of all the processes which nature has instituted for the purpose of protecting itself against the consequence of inflammation, there is none which exercises a more important office than that which induces the buffy coat in the blood: by it the progress of inflammation is arrested or circumscribed, by the deposition of coagulable lymph; we, therefore, find that, as soon as the system has time to sympathize, the buffy coat is developed; but it appears that a certain time is necessary for the accomplishment of this object: thus, blood drawn at the commencement of inflammation may not exhibit it.

I have often observed this circumstance, but more particularly in a case of pneumonia: in it, as long as the system appeared to be prevented from acting with freedom, owing to congestion of blood in the lungs, no buff was observed. I abstracted two pounds in the morning, and the same quantity during the same evening, with manifest advantage, but without finding buff. In the course of the next day, having deemed it necessary to have recourse once more to phlebotomy, the blood exhibited the strongest indication of inflammation, as well by the presence of the buffy coat, as by what is termed its cupped appearance. †

* Doctrine Homœopathique of Hahnemann, translated into French by Jourdan, p. 27.

† Having submitted the above to the venerable and highly experienced Dr. Storer, of Nottingham, the author is happy to have it in his power to give the valuable approbation of this learned physician in the following terms:

"*Lenton-firs; 7th February*, 1831.

"Dear sir: I am particularly pleased with your theory of the final purpose of the buffy coat in the blood produced by inflammatory action. So much is the human mind attached to the doctrine of causation, that any doctrine promising the solution of a phenomenon hitherto unaccounted for, gives it delight. In this theory you

This case would shew that, although, when the buffy coat is exhibited by blood drawn in diseases accompanied by other unequivocal symptoms of inflammation, we are to regard its presence as an absolute proof of the existence of that disorder, either in an acute or chronic form; at the same time its absence, under similar accompanying circumstances, should not lead us to form a contrary conclusion.*

Nature, in the developement of the buffy coat, not only furnishes itself with means of resisting disease, but also of assisting most materially in the operation of perpetuating our species. In the state of pregnancy, though a natural and healthy process, we observe many of the phenomena characteristic of inflammation. In it we observe acceleration of the pulse and local distention, attended, generally speaking, with marks of plethora. We also see the absorbent vessels called into action to detach the ovum, and next we find the system arming itself with coagulable lymph, in what may be called a free state, as is exemplified by the buff exhibited when blood is taken from pregnant women. A very little consideration will, however, serve to convince us that this peculiar state is highly necessary to enable the system to supply coagulable lymph, which is indispensable, as well for the adhesion of the ovum to the uterus, on its arrival within its cavity, as to provide for the gradual and daily increase of the fœtus, by the fixation (if I may use the expression) of the free lymph during the developement of its various parts. Thus we see that the presence of the buffy coat in the blood is only the effect of a salutary effort of the system, either to resist disease to repair its consequences, or to act a most important part in that most essential process of the animal economy, the perpetuation of our species.

It will also have been seen that, although its existence, when accompanied by other marks of inflammatory action, proves the surest and best test of the propriety of exercising sanguineous depletion; yet its absence, when other symptoms of this acute affection manifest themselves, ought not to deter us from having recourse to that most efficient remedy.

have analogy altogether on your side. We have abundant proof, in a variety of instances, that the laws of life are adjusted to the exigencies of disease, so as to obviate or compensate injuries otherwise inevitable: I see you have availed yourself of these instances. I know a lady of so delicate and debilitated a habit that, during her life, she could never bear the loss of an ounce of blood by a leech, without suffering much for weeks afterwards; on being seized with a pleuritic attack, she bore three full bleedings with relief, the two last buffed and cupped, and recovered well.

"Accept my thanks and best regards, and believe me to be, dear sir, yours, very truly, "John Storer."

* The greater part of these remarks on the buffy coat in the blood are taken from a paper of mine on the subject, published in the London Medical and Physical Journal for November 1829; but are again introduced here, to complete the present paper on the Theory of Febrile Action, &c.

Having thus far considered the nature and effect of that power of the system exemplified by the developement of the buffy coat in the blood, or what may be termed the material for reparation and reproduction, let us now take a view of a process which, though it appears to be antagonist to the one just described in its mode of action, is its great ally in attaining their ultimate object; I mean the increased action of the absorbents in removing superfluous matter, or even in inducing ulceration, where nature may wish to insulate a part or relieve local determination, by allowing effusion to take place from parts labouring under congestion. It would seem that nature, in its preservative efforts, is influenced by a species of instinct, arising out of an association of sensations which calls her resources into action, in a certain order of succession; and which, though it does not always confine them to the accomplishment of what is absolutely necessary and useful, like all the other laws of Providence, it was instituted for the general good of mankind. As soon as tension or swelling takes place, and the sensation it causes is experienced, the absorbents are called upon to act; soon after which provision is made in order to be in readiness to reconstruct what they may have carried away, by the developement of the buffy coat in the blood. We have a curious example of this even in some cases of dropsical swellings: distention* in such

* Distention to a certain extent exerts the same stimulating influence on the absorbents as pressure is known to do; but, if it be increased immoderately, instead of exciting, it paralyses their action. In this way we can account why general bleeding, by lessening the volume of the circulating mass, and thereby relieving over-distention, may prove apparently, and in some cases really, serviceable in certain cases of dropsy.

Magendie drew conclusions of the same practical utility, but from another chain of reasoning: he supposed that the action of the absorbents was diminished by plethora, and vice versâ. Thus, when he induced artificial plethora, by the injection of water into the veins, he could apply poisons to the pleura and stomach almost with impunity; while, on the contrary, when he bled the subject of his experiments so as to diminish the circulating mass, it very soon fell a victim to the effects of poisonous absorption. Magendie's experiments are only further proofs of the wisdom with which nature has instituted general laws for the preservation of life. It appears very natural that, when the vessels are distended with water, or that plethora exists, she must feel conscious of the impropriety of increasing the action of the absorbents, and vice versâ. On the same principle, we see the lungs increase or decrease their consumption of oxygen in the formation of carbonic acid gas, according to the nature of our aliment. Dr. Paris, in his Pharmacologia, vol. i., sixth edition, p. 213, says, "Mr. Spalding, the celebrated diver, found that when he ate animal food, and drank spirits, he consumed in a much shorter time the oxygen in his diving bell, and therefore confined himself to water and vegetables when following his profession. Nature in this case exercises her discriminating powers, arising out of an association of sensations, and finding that animal food and spirits contained less oxygen than vegetables and water, induces the lungs to endeavour to extract from the atmosphere more oxygen when the system is supported on the former than on the latter kind of diet. A knowledge of this circumstance is essential, in order to enable us to explain the rationale of our dietetic regulations in disease of the respiratory organs.

instances, is generally supposed to be caused by an increase of action in the exhalent vessels, to counteract which the absorbents are naturally called upon to use their endeavours to correct the effect of this deviation from healthy action: by association, their ally, the buffy coat, is invited to come to their assistance; and hence may have arisen the idea that dropsy depends on an inflammatory state of the system in general.

But to return to our subject of inflammatory fever: as I already stated, if its causes have been slight, its duration will be ephemeral; but should they be intense, the efforts of nature may be unable to re-establish the lost equilibrium within a short period of time; owing to which, it may assume some of the characters of remittent fever; in which case, the treatment, of which an outline has been given, should be persevered in, though with modifications according to the circumstances of the case. The shades of difference between the condition of the blood in various individuals, from that which exists in the most vigorously constituted, to that which borders on putrescency and is formed in particular habits, are very numerous, and influence in a proportionate degree the gradations in the complexion of the diseases which assail mankind.

We even find fever gradually change its form, in some instances, from being purely inflammatory to the remittent and intermittent type; owing to the change which is induced in the state of the blood during disease, unfitting it to continue the sthenic efforts which it had commenced. It is quite evident to all medical observers that the changes in the form of disease take place according to the condition of the circulating fluids; on the same principle that the same morbific causes will induce disease of different forms in different individuals whose condition of blood, or what may be termed predisposition, varies. When quartered in the West Indies, I witnessed a striking example of this fact. Owing to the influence of a small artificial swamp which formed to windward of a building, eight persons were attacked with fever; in some the disease was of a highly inflammatory nature; in others, yellow fever of a typhoid type manifested itself, and one individual had ague; so that each was affected according to previous predisposition. The young and vigorous were those who suffered from inflammatory fever; the elderly and drunken from the typhoid disease; and a delicate but temperate woman was the subject of ague. I say these different forms of disease all arose from the same source; because this building was isolated, and the troops in other parts, though not far distant, were healthy; and further that, as soon as the cause was discovered and removed, no fresh case appeared. It may also be well to remark, that the cases alluded to, some of which were fatal, were taken to hospital, and placed indiscriminately with other white patients, where they were attended by white orderlies, the hospital sergeant, and myself, without affording a single instance of the propagation of the disease.

I am of opinion that the high temperature of a West India climate must so rarify and separate the atoms or ultimate particles of which emanations from animal bodies are composed, as to deprive them of their virulence; more particularly, when due ventilation and cleanliness are attended to, as is the case in all of his Majesty's hospitals: for the same reason we observe that miasmal exhalations are comparatively innoxious during the heat of the day, while they are highly destructive in their effects both immediately before sunrise and soon after sunset; hence the danger of sleeping on the ground or bivouacing in a tropical climate; or of exposure, under any circumstances, before the sun has sufficiently risen. The treatment of asthenic fevers must be conducted with a view to the correction of the disordered state of the fluids and the support of the strength generally: bark, arsenic, iron, &c., act specifically on the nervous system by arresting the paroxysms or exacerbations of fever, and afford instances of the direct influence of medicine in its action on the solids. The consideration of these effects, while it points out the mode which providence has given us of checking nature's efforts when inordinate will, at the same time, serve to explain the impropriety of treating diseases on principles of solidism alone. We find by experience that it is bad practice to endeavour, in all states of fever, to arrest paroxysmal efforts, which, we are aware are instituted to rid the constitution of the impurities with which the blood becomes charged, and to re-establish equilibrium between the nervous and vascular systems.

The untimely and consequent empirical exhibition of bark has, at various epochs since the discovery of that invaluable remedy, brought it into disrepute, and not undeservedly, as, by arresting the paroxysms of fever without having previously improved the state of the fluids, by exciting the excretory organs to carry off the noxious principles accumulated in the blood, we only lock up the wolf in the sheepfold, and interfere with nature in her endeavours to restore health:* on the contrary, in order to imitate her in her steps, without being a bigot in favour of the humoral pathology, or exclusively devoted to that of solidism, we ought first to endeavour to improve the condition of the circulating fluids by evacuants and alteratives, while we give due attention to the strength of the system, and support it, if necessary, by tonics, &c., and then, but not till then, direct our views to arrest the paroxysmal disposition by medicines which act directly on the solids. Such will be found to be the most rational and effective method of treating all paroxysmal affections, and that which will cut them short, and remove disease at the least possible expense to the constitution.

* "This is a very important and valuable practical precept; and, being in opposition to many high authorities, I find it more necessary to declare that the opinions here advocated are firmly held by me, after a practice of upwards of half a century. J. S."

The above note was added to the manuscript of this publication when the author submitted it for Dr. Storer's perusal and opinion.

Tic douloureux, and the great variety of inexplicable nervous disorders belonging to the same class, ought to be treated on the same general principles, as, however efficacious arsenic, iron, or quinine, may be deemed in arresting them, they will generally fail to induce a permanent cure, if not preceded or accompanied by a proper attention to the state and quality of the secretions and blood generally. It may in some cases of fever, when the causes are intense and the constitution in a bad state, be necessary, for the preservation of life, to cut short the paroxysmal action by acting quickly on the solids; but, if so, the state of the fluids must not be neglected, or the relief would be only temporary. Of all the forms in which bark has been administered with most success for the cure of ague, that in which it has been combined with alteratives, laxatives, and deobstruents, has been the most so; such a combination will, at the same moment, excite the liver, intestines, kidneys, &c. to rid the blood of noxious principles, while it acts specifically on the solids by arresting febrile paroxysms: by this combination of effects disease is stopped, and nature is saved the trouble and expense of making additional efforts for the purification of that vital stream, the blood. In remittent and continued fever of a typhoid type, in which the alkaline treatment recommended by Dr. Stephens and others has proved so efficacious: the addition of quinine to the doctor's plan will be found to increase its utility in an eminent degree; indeed, I know of no medicine so decidedly febrifuge as quinine, when it is administered at the proper stage of the complaint. It may be conveniently given in a state of effervescence, by adding the quinine to the tartaric acid solution, in which it dissolves readily; an excess of soda may then be prescribed in the alkaline solution, either with or without a given quantity of the Tart. Sodæ et Potassæ, according to the state of the alvine evacuations. This combination acts equally on the fluids and solids, and will, by exciting the secretions, and thus purifying the blood, while the quinine acts directly on the solids, restore in the most direct manner the equilibrium of tone between the vascular and nervous systems, and with it health.

This view of the *methodus medendi* will, I trust, be found to be the most philosophical and effectual which can be taken with regard to the treatment of every species of paroxysmal disease, whether it presents an acute or chronic form; and will, also, be found to be that followed virtually by the most judicious practitioners, although they may not explain its rationale on the same principles.

I think it unnecessary to extend this essay more, although it might be made, without difficulty, to fill a large volume, as, in the present enlightened state of the medical world, the general principles which I have ventured to submit to it will, I trust, enable practitioners to apply them efficaciously to the treatment of diseases, whether they be local, general, or vicarious; while they will also have explained the futility and even dangerous consequences of founding systems of medical practice on the exclusive physiological doctrine of either humoralism or solidism.

I cannot, however, conclude without expressing my grateful acknowledgments to Dr. Storer, of Nottingham, to whom I submitted the manuscript of this essay; and who was kind enough to afford me the benefit of his well-known taste and almost unexampled experience in its revision, and whose approbation likewise induces me with more confidence to lay it before the profession.

*Nottingham; November* 1832. (Continued vol. lxxx p. 15.)

